# A negative effect of *Campylobacter* capsule on bacterial interaction with an analogue of a host cell receptor

**DOI:** 10.1186/1471-2180-14-141

**Published:** 2014-05-31

**Authors:** Sona Rubinchik, Alan M Seddon, Andrey V Karlyshev

**Affiliations:** 1 School of Life Sciences, Kingston University, Faculty of Science, Engineering and Computing, Penrhyn Road, Kingston-upon Thames KT1 2EE, UK

**Keywords:** Campylobacter, Adhesion, Attachment, Adhesins, Receptors, Glycoproteins, Glycosylation, MGL receptor, C-type receptor, Lectins

## Abstract

**Background:**

*Campylobacter jejuni* (*C. jejuni*) is the leading causative agent of bacterial gastrointestinal infections. The rise of antibiotic resistant forms of this pathogen necessitates the development of novel intervention strategies. One approach is the design of drugs preventing bacterial attachment to host cells. Although some putative *C. jejuni* adhesins have been identified, the molecular mechanisms of their interaction with host cells and their role in pathogenesis remain to be elucidated. *C. jejuni* adhesion may also be modulated by a bacterial capsule. However, the role of this structure in adhesion was not clear due to conflicting results published by different research groups. The aim of this study was to clarify the role of capsule in bacterial interaction with host cells by using an *in vitro* model of adhesion and an analogue of a host cell receptor.

**Results:**

In this study, we developed an *in vitro* bacterial adhesion assay, which was validated using various tests, including competitive inhibition studies, exoglycosydase treatment and site-directed mutagenesis. We demonstrate that PEB3 is one of the cell surface glycoproteins required for bacterial interaction with an analogue of a host cell receptor. In contrast, JlpA glycoprotein adhesin is not required for such interaction. We demonstrate that the production of capsule reduces bacterial attachment, and that the genes involved in capsule and PEB3 adhesin biosynthesis are differentially regulated.

**Conclusions:**

In this study we report an *in vitro* model for the investigation of bacterial interaction with analogs of host cell receptors. The results suggest an interfering effect of capsule on bacterial attachment. In addition, using a liquid culture, we demonstrate differential expression of a gene involved in capsule production (*kpsM*) and a gene encoding a glycoprotein adhesin (*peb3*). Further studies are required in order to establish if these genes are also differentially regulated during the infection process. The results will assist in better understanding of the mechanism of pathogenesis of *C. jejuni* in general and the role of capsule in the process in particular.

## Background

*Campylobacter jejuni (C. jejuni*), a microaerophilic, spiral-shaped, flagellated Gram-negative bacterium, is the most frequent cause of human gastroenteritis worldwide [1]. *C. jejuni* infections are often caused by consumption of undercooked poultry, unpasteurised milk or contaminated water [2]. Adhesion of *C. jejuni* to host cells plays an important role in colonisation of chickens and in human infection [3]. *Campylobacter* binding to host cell receptors is not mediated by fimbria or pili, like in *E. coli* and *Salmonella*[4]. As noted in a recent review, other bacterial cell structures may contribute to interaction of *Campylobacter* with host cells [5].

In some cases, bacterial adhesion can be mediated by oligosaccharides present on the surface of host cells [6,7]. In other cases, it is a pathogen oligosaccharide that is responsible for binding to specific, lectin-like, host cell structures. For example, a pathogenic Gram-positive bacterial species *Nocardia rubra* binds to a human lectin (intelectin) expressed by cells in different organs including intestine [8]. There is a possibility of similar lectin-glycan interactions between host cells and *Campylobacter*. For example, *C. jejuni* adhesion to Caco-2 cell receptors was inhibited by certain lectins [9].

*Campylobacter* is capable of producing a variety of glycoproteins, some of which are cell-surface located [10]. Inactivation of the N-linked glycosylation system reduces bacterial ability to adhere to epithelial cells and thereby colonise the gastrointestinal tract [11,12]. These findings suggest a possible role of some bacterial cell surface surface-located bacterial N-linked glycoproteins in interaction with host cell receptors. Van Sorge and colleagues [13] demonstrated interaction of N-linked glycoproteins of *C. jejuni* with C-type lectins of Macrophage Galactose-type lectins (MGL). In similarity with other pathogens, the production of cell surface structures interacting with C-type lectins may assist *C. jejuni* in the evasion of the host immune response [14,15].

Another cell surface structure that may affect bacterial interaction with host cell receptors is a capsular polysaccharide (CPS) [16-19]. Inactivation of the capsule production machinery in strain 81–176 led to a two-fold decrease in adhesion to INT407 cells [20]. Similar findings were observed in another capsule deficient mutant, 81116/*kpsE*[21]. However, these data were not supported by complementation studies. Moreover, they are in disagreement with other studies where the absence of capsule showed increased adhesion of *C. jejuni* strain 11168H to Caco-2 cells [16]. The contradictory results may be a consequence of differences in assay conditions, bacterial strains and tissue cell lines.

In general, the capsules may play different roles in bacterial attachment. This depends on the nature of a bacterial pathogen, and on the structural features of the capsules and adhesins. For example, F1 capsule of a *Yersinia pestis* prevents fimbrial adhesins from interaction with host cell receptors [22], while production of a capsule by *Neisseria meningitidis* does not affect PilC1 adhesin-mediated bacterial attachment [23].

In this study we developed and evaluated an *in vitro* ELISA-like assay for the investigation of *C. jejuni* interaction with host cell receptors. The assay was successfully used to study a role of capsule in attachment using SBA (Soya bean agglutinin) lectin as an analogue of a host cell receptor. In addition, using targeted mutagenesis (supported by complementation analysis) we investigated a role of PEB3 and JlpA adhesins in this interaction. Furthermore, using real time PCR, we found that *peb3* and a capsule-related gene are differentially expressed. The results of these experiments suggest an interplay between bacterial capsule and adhesins in interaction with host cells.

## Results

### Dose-dependent specific binding of *C. jejuni* cells to immobilised SBA lectin

In order to investigate the mechanisms and factors involved in *C. jejuni* interaction with host cells, we developed an ELISA-like assay based on monitoring bacterial binding to immobilised analogues of host cell receptors. In this model we explored the idea that some N-link glycosylated proteins may be expressed on the bacterial cell surface, and may potentially play a role of adhesins. As glycan moieties in these glycoproteins contain terminal GalNAc residues recognised by SBA, we used the latter as an analogue of a host cell receptor.

**Figure 1:**
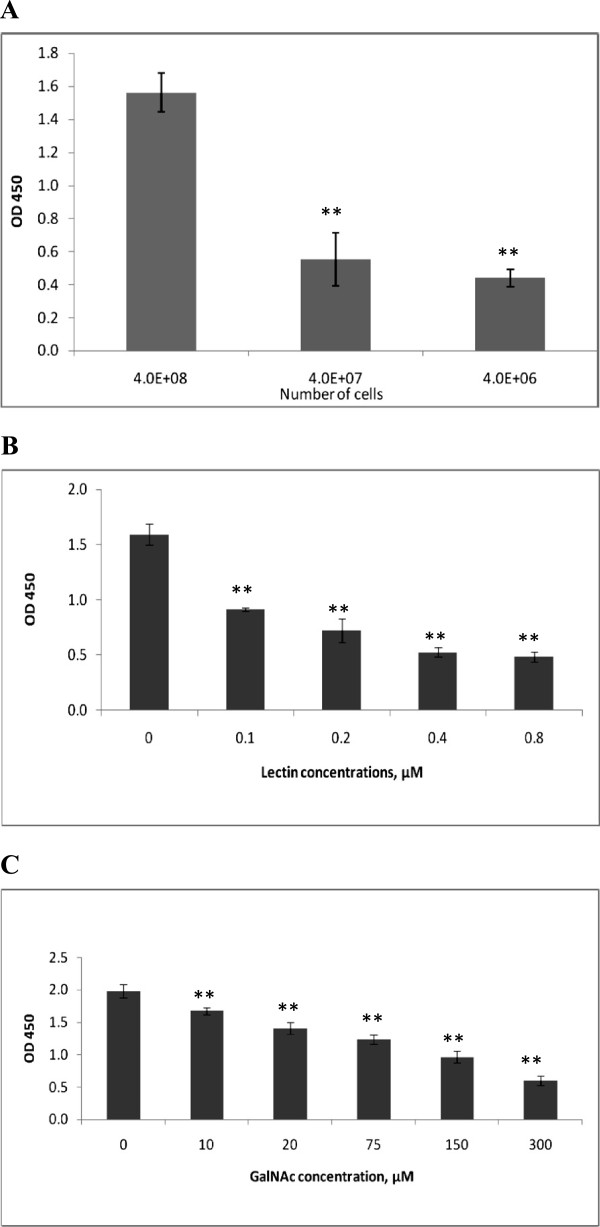
**Interaction of *C. jejuni *with immobilised SBA. (A) ***C. jejuni* 11168H interaction with SBA lectin is concentration dependent. The figures below the bars indicate the numbers of cells per well. **(B)** Effect of different concentrations of soluble SBA lectin on binding of *C. jejuni* 11168H. **(C)** Effect of different concentrations of GalNAc on binding of *C. jejuni* 11168H.

**Figure 2:**
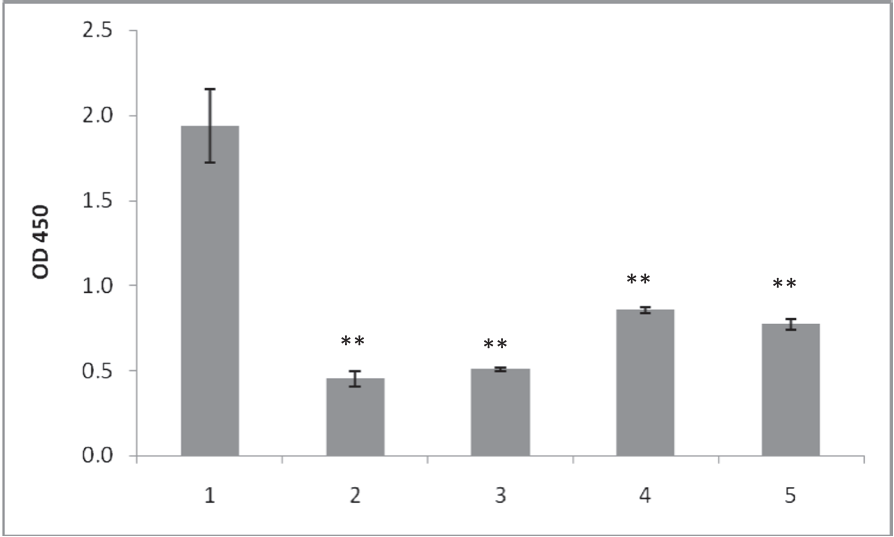
**Detachment of cells of *C. jejuni *** **11168H in the presence of 5 mM and 10 mM of soluble lectin (2 and 3 respectively), or 5 mM and 10 mM of GalNAc (4 and 5 respectively).**

**Figure 3:**
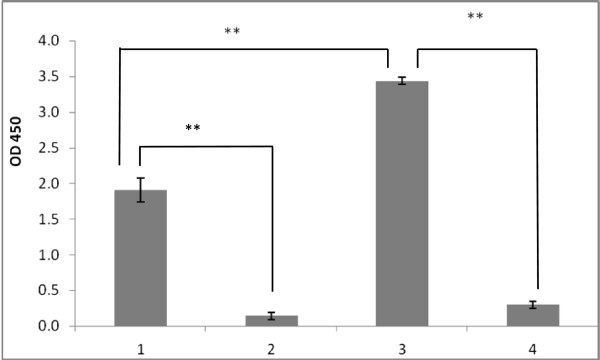
**Reduction of binding upon treatment of bacteria with GalNAc-specific exoglycosydase.** Results with *C. jejuni* 11168H strain (1 and 2) and its isogenic non-capsulated mutant 11168H/*kpsM::kan*^*r*^ (3 and 4) are presented. Samples before (1 and 3) and after (2 and 4) treatment with exoglycosidase are shown. Elimination of capsule increases bacterial attachment (1 and 3).

Incubation of a suspension of *C. jejuni* 11168H cells with immobilised SBA resulted in bacterial attachment (Figure 1A). This binding was found to be specific as demonstrated by inhibitory effects by both GalNAc and a soluble form of SBA in a dose-dependent manner. The inhibitory effect was detectable with as low concentration of SBA lectin as 0.1 μM (Figure 1B). GalNAc also showed an inhibitory effect at concentrations over 10 μM (Figure 1C). Moreover, the bound cells could be detached in the presence of a soluble form of lectin or GalNAc (Figure 2). Further confirmation of specific binding was obtained by treatment of bacterial cells with an exoglycosidase. Removal of a terminal GalNAc resulted in a remarkable reduction of the ability of bacterial cells to attach (Figure 3)*.*

**Figure 4:**
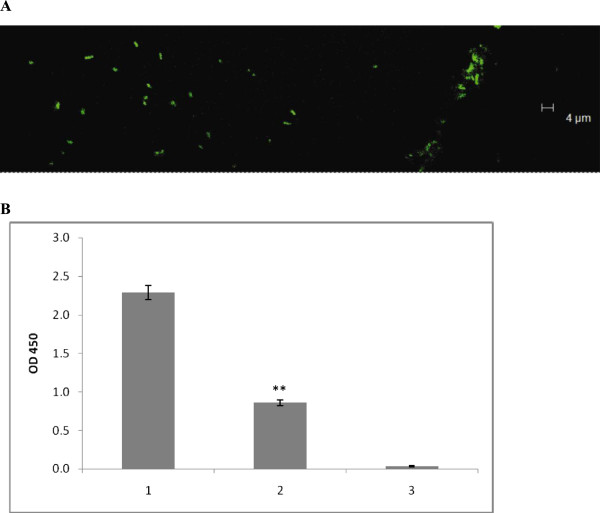
**Interaction of *E. coli *cells, containing *C. jejuni *glycosylation gene cluster, with SBA lectin. (A)** Confocal microscopy of *E. coli* XL2/pPGL1 after treatment with fluorescently labelled SBA. No fluorescence was observed for *E. coli* XL2 control (data are not shown). **(B)** Attachment of *E. coli* XL2/pPGL1 to immobilized SBA lectin (1) is inhibited by GalNAc at 5 mM (2). No binding of the recipient strain *E. coli* XL2 was detected (3).

In order to further confirm that the developed model of attachment is specific and is based on the surface-located GalNAc moieties, we repeated the binding experiments using *E. coli* cells carrying the entire N-linked protein glycosylation apparatus (*pgl* gene cluster) of *C. jejuni*[24]. Due to the absence of glycosylation acceptor proteins in strain *E. coli* XL2/pPGL1, the *pgl* system was found to be able to glycosylate the bacterial lipo-polysaccharide, resulting in exposure of GalNAc residues on the cell surface [24] (Figure 4A). The results confirmed that *E. coli* XL2/pPGL1 cells are capable of binding to immobilized SBA lectin in a GalNAc dependent fashion (Figure 4B).

### Expression of PEB3 is required for binding of *C. jejuni* cells to immobilised SBA lectin

**Figure 5:**
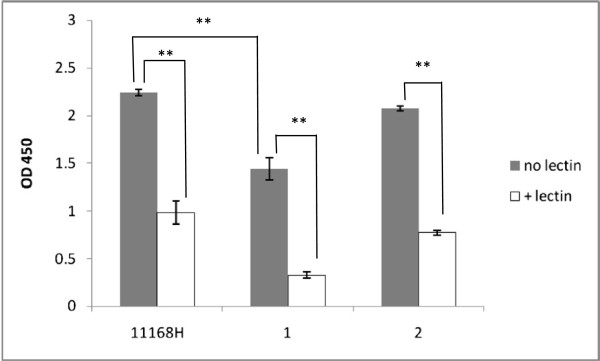
**Insertional inactivation of gene *peb3 *reduced the ability of strain 11168H to bind immobilised lectin.** 1, recipient (11168H); 2, mutant (11168H/*peb3::kan*^*r*^ ); 3, complementation derivative (11168H/*peb3::kan*^*r*^*/peb3+*).

Previous studies suggested a possible location of PEB3 protein on a bacterial cell surface [25,26]. The purified PEB3 protein was able to bind SBA lectin due to the presence of a GalNAc-containing glycan moiety [26]. In order to confirm that attachment of *C. jejuni* cells to immobilised SBA in our experiments is mediated by PEB3, we constructed and investigated the binding properties of the respective mutant. The results demonstrated significant reduction of attachment of 11168H/*peb3::kan*^*r*^, which was restored after complementation (Figure 5).

The results of this experiment also showed that *peb3* mutation did not completely eliminate binding, suggesting that other glycoprotein(s) may be involved in specific interactions with this analogue of a host cell receptor. This hypothesis was supported by reduction of the residual binding of 11168H/*peb3::kan*^*r*^ mutant in the presence of soluble lectin (Figure 5).

One of the other cell surface-located proteins of *C. jejuni* is JlpA, which was found to be an adhesin specifically binding to heat shock protein 90 [27]. As JlpA was also predicted to be an N-link glycosylated protein [28], there was a possibility that it might be responsible for residual binding of 11168H/*peb3::kan*^*r*^ mutant. To verify this hypothesis, we constructed a *jlpA* mutant and tested the effect of this mutation on attachment. Surprisingly, none of the three independent clonal isolates showed any difference when compared with the control recipient strain 11168H (data not shown) suggesting the presence of other GalNAc-containing adhesins.

### Production of capsule has a negative effect on binding

The results shown in Figure 3 also have demonstrated a significantly higher efficiency of binding of the non-capsular mutant of strain 11168H. These results, confirmed by analysis of three independent clonal isolates of this mutant (data not shown), revealed significant increase in binding upon inactivation of bacterial ability to produce capsule, suggesting an interfering effect of the later on the bacterial interaction with host cell receptors.

### *Peb3* and capsule-related genes are differentially expressed

**Figure 6:**
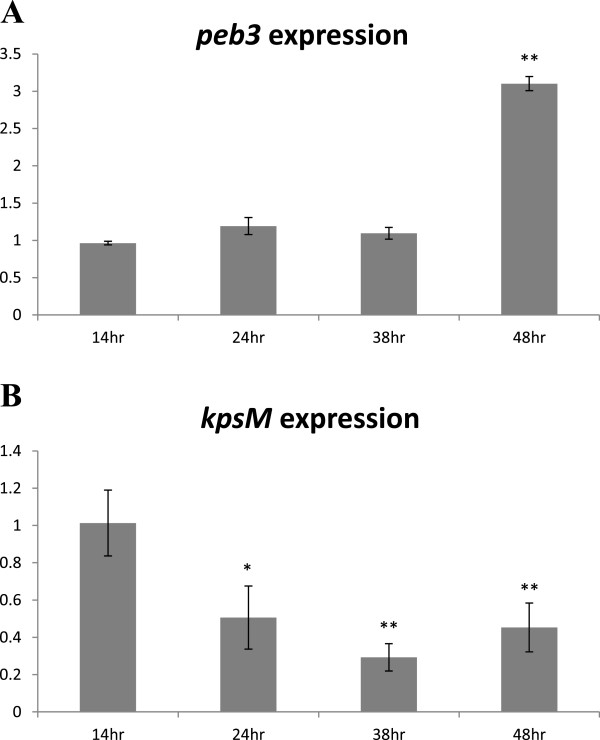
**Increase of peb3 gene expression (A) and decrease of kpsM expression (B) over time in a liquid culture.** Gene expression levels relative to 16S rRNA were determined as described in Materials and Methods section.

Due to antagonistic effects of capsule and PEB3 adhesin on bacterial attachment, we hypothesized that these structures might be differentially expressed. To test this hypothesis we conducted a comparative analysis of the dynamics of *kpsM* and *peb3* gene expression at different growth stages in a liquid culture using real time PCR (RT-PCR). The results showed a dramatic increase in the level of *peb3* gene expression at 48 hrs (Figure 6A), whilst there was a reduction in *kpsM* gene expression after 24 hrs (Figure 6B).

## Discussion

In this study, a model of bacterial attachment was developed. This model is based on monitoring bacterial binding to immobilized analogues of host cell receptor. Although we only tested attachment of *Campylobacter jejuni* to SBA lectin, the method may have wider application for investigation of interaction of other bacteria with other host cell receptors and their analogues.

The system was successfully tested by using *C. jejuni* strain 11168H and its isogenic mutant 11168H/*peb3*. Using the assay, we investigated interaction of bacteria carrying cell surface located GalNAc residues with immobilised SBA lectin. The binding was found to be specific and dependent on the presence of soluble lectin and GalNAc molecules, and was abolished by bacterial deglycosylation. The study suggests the ability of *C. jejuni* to produce various cell surface GalNAc-containing cell surface structures.

The SBA lectin used in this study shares binding specificity with C-type lectins (including MGL receptors) produced by host cells. According to a recent study, *Campylobacter* has the ability to interact with MGL receptors expressed on macrophages and dendritic cells (DCs), which may modulate host immune response [13]. Human MGL receptors specifically recognise terminal GalNAc residues [29,30]. Together with other C-type lectins, the MGL receptors may be recognised by viruses, e.g. a filovirus [31]. In addition, it was shown that MGL recognizes a GalNAc containing antigen of a helminth parasite *Shistosoma mansoni*[32]. Despite some data suggesting a role of MGL receptors as a host defence factor, the role of these molecules in *C. jejuni* infection is not clear. However, there is a possibility that, via interaction with MGL expressing macrophages and DCs this pathogen may subvert host immune response. It was suggested that *C. jejuni* with functional MGL ligand (GalNAc) may decrease IL-6 production by DCs [13].

*Campylobacter* have been known to produce a number of N-glycoproteins, including PEB3 [33]. However, it was still unclear which glycoprotein is reactive with MGL. Our results demonstrated that *peb3* mutation reduces but does not completely eliminate binging, suggesting the presence of other cell surface structures responsible for attachment. Surprisingly, mutation in *jlpA* gene, encoding another cell surface glycoproptein, had no effect on the ability of *C. jejuni* to bind to the immobilized SBA lectin. According to other studies, *jlpA* mutation also had no effect on invasion of host cells [34,35].

An isogenic non-capsulated mutant 11168H/*kpsM* showed an increase in binding indicating a negative effect of capsule on bacterial attachment. In addition, results of RT-PCR showed an increase of *peb3* and a decrease of *kpsM* gene expression over time, suggesting that a shielding effect of capsule may be essential at the initial stages of infection, hiding bacterial cell surface structures. Subsequent down regulation of CPS production during colonisation may lead to exposure of other bacterial cell surface structures required for the attachment and/or evasion of host immune response.

## Conclusions

The results of this study demonstrated a complex interplay of *Campylobacter* capsule and glycoprotein adhesins in pathogen-host interaction. The developed assay will assist in more detailed investigation of such interaction and in the development of inhibitors of attachment as novel antibacterials.

## Methods

### Bacterial strains and growth conditions

*C. jejuni* strain 11168H and its isogenic mutant 11168H/*kpsM::kan*^*r*^ were described previously [19,36]. *C. jejuni* was grown under microaerophilic conditions (5% O_2_, 10% CO_2_, 85% N_2_) at 37°C on Columbia Blood Agar (Oxoid) containing 6% defibrinated horse blood (Fisher) and Skirrow supplement (Sigma). Antibiotics (chloramphenicol 10 μg/ml and/or kanamycin 50 μg/ml) were added to the media as required. *E. coli* strains XL1 and XL2 (Stratagene) were used in cloning experiments. *E. coli* strains were maintained on Luria–Bertani agar (Oxoid) plates or in Luria–Bertani broth (Oxoid) supplemented with appropriate antibiotics (ampicillin 100 μg/ml, kanamycin 50 μg/ml or chloramphenicol 34 μg/ml) at 37°C.

### General cloning techniques

Molecular cloning was performed using standard protocols. The plasmids used in this study are listed in Table 1. Restriction enzymes and antarctic phosphatase were purchased from New England Biolabs. T4 DNA ligase and T4 DNA polymerase were purchased from Promega. Oligonucleotides were ordered from Sigma-Genosys. Genomic and plasmid DNAs were extracted using Qiagen kits. Restriction, DNA ligation, dephosphorylation and blunt-ending were performed according to manufacturers’ protocols.

**Table 1:** Plasmids used in this study

***Plasmids***	***Description***	***Source (reference)***
pGEM-T Easy	Cloning vector	Promega
pJMK30	Source of *kan*^*r*^ cassette	[37]
pAV35	Source of *cam*^*r*^ cassette	[37]
pBAD33	Contains pBAD promoter	[38]
pPGL1	*C. jejuni* 16 kb fragment, containing *pgl* gene cluster, cloned into pBR322	[24]
pRRC	Cassette cloned into pRR (fragment of rRNA gene cluster cloned into pGEM-T easy)	[39]

### Construction of *C. jejuni* mutants

Fragments of the genes *peb3* and *jlpA* were PCR amplified using the primers listed in Table 2 and cloned into pGEM-T Easy (Promega) vector to produce plasmids pGEM_peb3 and pGEM_jlpA respectively. In order to disrupt the *peb3* gene, the pGEM_peb3 plasmid was digested with *Pfl*MI, blunt ended and ligated with the *Sma*I-digested *kan*^*r*^ cassette producing pGEMpeb3_kan construct. The *jlpA* gene was disrupted by insertion of the *Xba*I-digested *cam*^*r*^ cassette (isolated from pAV35, Table 1) into the *Bsa*BI restriction site of pGEM_jlpA plasmid*,* yielding pGEM_jlpA_cam*.* In both cases the orientation of the antibiotic resistance cassette was the same as that of the target gene to avoid a negative polar effect in the mutants. Mutagenesis using the constructed derivatives was conducted via electroporation and selection of the derivatives on media supplemented with appropriate antibiotics. Allelic replacement was confirmed by PCR. The mutants were designated 11168H/*peb3::kan*^*r*^ and 11168H/*jlpA::cam*^*r*^*.*

**Table 2:** **Primers used for mutation of *peb3 *** **and *jlpA *** **and for complementation of *peb3***

**Primer**	**Sequence (5′-3′)**	**Used for**
peb3_for	ATGAAAAAAATTATTACTTTATTTGGTGCATG	Mutation of *peb3* gene
peb3 _rev	TTATTCTCTCCAGCCGTATTTTTTAAAAATTTC	Mutation of *peb3* gene
jlpA_for	ATGAAAAAAGGTATTTTTCTCTCTATTGG	Mutation of *jlpA* gene
jlpA_rev	TTAAAATGACGCTCCGCCCATTAACATAG	Mutation of *jlpA* gene
peb3_XbaI_for	ATAATCTAGAAAGGAAATACTATGAAAAAAATTATTACTTTATTTGGTGC	Complementation of *peb3* mutation
Peb3_XbaI_rev	AGGTTCTAGATTAATGATGATGATGATGATGTTCTCTCCAGCCGTATTTTTTAAAAATTTC	Complementation of *peb3* mutation

### Complementation of *peb3* mutant

*Peb3* gene was PCR amplified using primers described in Table 1. The product was digested with *Xba*I enzyme and cloned into *Xba*I-digested pRRC plasmid to produce pRRC_peb3. Restriction analysis verified that the gene was transcribed in the same orientation as the *cam*^*r*^ gene. After transformation of the 11168H/*peb3::kan*^*r*^ mutant with plasmid pRRC_peb3, Kan^r^Cam^r^ clones were selected. PCR analysis confirmed integration of *peb3* gene into one of the rRNA gene clusters. The complementation derivative was designated 11168H/*peb3::kan*^*r*^*/peb3*^*+*^*.*

### Binding assay

Bacterial attachment was studied in ELISA-like assay using a 96-well microtiter plate Maxisorp™ (Thermo Scientific) coated Soya Bean Agglutinin (SBA) lectin (Sigma) in bicarbonate-coating buffer: 5.3 g/L Na_2_CO_3_, 4.2 g/L NaHCO_3_, 1 g/L sodium azide, pH 9.6.

Microtiter plate wells were incubated overnight with SBA lectin (10 μg/ml) at 4˚C, followed by blocking with 1% Bovine Serum Albumin (BSA) overnight at 4°C. BSA-coated, wells were used as negative control. Bacteria (two-day cultures of *C. jejuni* or one-day cultures of *E. coli*) were harvested, resuspended in Phosphate-Buffered Saline (PBS) to OD_600_ = 1, 0.1 ml suspensions (corresponding to 4x10^8^ c.f.u. of *C. jejuni*) were added to each well of the microtiter plate, followed by incubation for 40 min at room temperature. After rinses with PBS, supplemented with 0.2% Tween (PBST) the plate was incubated with biotinylated SBA lectin (Vectors Laboratories) for 60 min at room temperature. The wells were then treated with horseradish peroxidase-conjugated streptavidin (Sigma) for 30 min at room temperature followed by incubation with TMB (3,3′,5,5′-Tetramethylbenzidine) substrate (Sigma) for 10 min. The reaction was stopped by adding stop solution (1 M H_2_SO_4_). Binding was monitored by measuring OD at 450 nm. For inhibition studies, the plates were treated with bacterial suspension in the presence of SBA lectin or GalNAc (Sigma) at different concentrations. Detachment was carried out by addition to wells with immobilised bacteria of either soluble SBA lectin or GalNAc, followed by incubation for 40 min at room temperature.

### Fluorescein SBA (FSBA) labelling of *C. jejuni* and *E.coli* cells

Fluorescein labelling of cells was done as described previously [40]. FSBA (Vector Laboratories) (100 μg/ml in PBS) was mixed with an equal volume of bacterial suspension and incubated for 40 min at room temperature. Bacteria were pelleted, washed twice in PBS to remove any unbound lectin. Samples were observed by fluorescence microscopy using a laser scanning confocal microscope (Leica TCS SP2 AOBS) with a 63X immersion objective.

### Treatment with exo-glycosidase

In order to remove GalNAc residues bacterial cells were treated with 20 U of *N-*acetylgalactosaminidase (NEB) for 60 min at 37°C according to manufacturer’s protocol.

### RNA isolation and RT-PCR

For RNA isolation, *C. jejuni* cells were grown for 48 hours under microaerophilic conditions (5% O2, 10% CO2, 85% N2) at 37° in three separate flasks (biological replicates) in Brain Heart Infusion Broth (Oxoid). Samples for RNA isolation were taken at 14 h, 24 h, 38 h and 48 h intervals. Immediately after taking the samples from the flasks RNAprotect Bacteria Reagent (Qiagen) was added to the cultures to stabilize mRNA. The total RNA from each sample was extracted using the RNeasy Mini Kit (Qiagen). The purified RNA samples were treated with On-Column DNaseDigestion Kit (Qiagen) followed by treatments with DNase in order to remove residual DNA contamination. RNA concentration was estimated using NanoDrop ND-1000 spectrophotometer (NanoVue). The quality and integrity of total RNA was monitored using the Agilent 2100 Bioanalyzer (Agilent Technologies).

RT-PCR was used for gene expression studies of *peb3* and *kpsM* using primers listed in Table 3. Primers were designed from *C. jejuni* DNA sequences using NCBI web server (http://www.ncbi.nlm.nih.gov/tools/primer-blast/). In addition, potential secondary structures and primer dimer formation were verified using an on-line tool, Sigma-Genosys DNA calculator. Primers were purchased from Sigma Genosys Ltd. One-step RT-PCRs were performed in triplicate by using QuantiFast SYBR Green RT-PCR Kit (Qiagen). The RT-PCR reaction was performed in a total volume of 12.5 μl, containing 6.25 μl master mix and 0.25 RT mix, consisting of 1 μl forward primer, 1 μl reverse primer 3.6 μl diluted RNA (50 ng) and 6.25 μl water. Primers were added to 100 μM final concentration. Each sample was analysed in technical duplicates and biological triplicates.

**Table 3:** Primers used for RT-PCR

**Primer name**	**Primer sequence (5′-3′)**	**Used for**
q-16 s-for	ACAGGTGCTGCACGGCTGTC	Control
q-16 s-rev	TGCTCGGCCGAACCGTTAGC	Control
Peb3_qpcr_for	GCATCGGCTTGATCTTGTGCG	*peb3* expression
Peb3_qpcr_rev	CGTTGTGCCTGAAGGTGCTGG	*peb3* expression
kpsM_qpcr_for	GCCTGAGTTCCATTCATAAGCTGGG	*kpsM* expression
kpsM_qpcr_rev	TGGGTAGTTGGGGAGCCTATGAG	*kpsM* expression

The cycle parameters were as follows: an initial reverse transcription step for 10 min at 50°C, and then PCR initial activation step for 5 min at 95°C followed by two-step cycling: denaturation at 95°C for 10 s and combined annealing 60°C for 30 sec. The number of cycles was 35. The changes in gene expression (*n*-fold) calculated from the qRT-PCR data. Analysis of relative gene expression data was done using the 2^-2∆∆*C*T^ method as described previously [41]. The 16S rRNA was used as the internal controls.

### Statistical analysis

All experiments were repeated a minimum of three times, and data are expressed as mean ± SD. Differences were considered significant for P < 0.05 (*, P value 0.05-0.01; **, P value <0.01). Comparison of two groups was made with an unpaired, two-tailed student’s t-test. Comparison of multiple groups was made with ANOVA.

## Competing interests

The authors declared that they have no competing interests.

## Authors’ contributions

SR carried out the experiments, analysed the data and was involved in manuscript preparation. AVK conceived and designed the study, was involved in setting up the experiments and data analysis, and prepared the manuscript for submission. AS was involved in coordination and design of the study, and in manuscript preparation. All authors read and approved the final manuscript.
